# Implementation of a Hot Gallbladder Service at a District General Hospital: A 15-Month Review

**DOI:** 10.7759/cureus.101972

**Published:** 2026-01-21

**Authors:** Xiangmei C Cui, Osama Zaman, Mushal Naqvi

**Affiliations:** 1 Surgery, Walsall Healthcare NHS Trust, Walsall, GBR; 2 General and Colorectal Surgery, Russells Hall Hospital, Dudley, GBR; 3 General Surgery, Walsall Manor Hospital, Walsall, GBR

**Keywords:** acute calculous cholecystitis, early cholecystectomy, gallbladder diseases and gallstones, gallbladder removal, gallstone cholecystitis, hot gallbladder pathway, laparoscopic cholecystectomy

## Abstract

Background

The National Institute for Health and Care Excellence, the Association of Upper Gastrointestinal Surgeons, and the Tokyo Guidelines advocate early laparoscopic cholecystectomy (LC) as the standard of care for patients presenting with acute cholecystitis (AC). This study evaluates the implementation of a hybrid Hot Gallbladder (HGB) pathway, incorporating both inpatient index admission and fast-tracked outpatient management at a district general hospital in the United Kingdom with a 550-bed capacity, analysing its impact on service efficiency, early surgery rates, and alignment with guideline standards.

Methodology

A retrospective review was performed over a 15-month period (January 2023 to March 2024) following the introduction of the HGB service. Patients presenting with acute biliary pathology were identified from the Surgical Acute Care Unit (SACU) database. Patients meeting the defined eligibility criteria were included. Data were cross-verified across the SACU database, the HGB service list, and electronic patient records to ensure accuracy before analysis. Patients were grouped into pre-HGB (n = 84) and post-HGB (n = 202) cohorts. Statistical analysis was performed using chi-square testing, with significance set at p-values <0.05.

Results

A total of 286 patients were included (80.4% female; mean age of 45 years). The most common diagnosis was biliary colic (62.2%), followed by AC (27.3%) and gallstone pancreatitis (10.5%). Introduction of the HGB pathway significantly increased the rate of early LC from 13.1% to 30.2% (χ² = 9.19; p = 0.0024). The complication and 30-day readmission rates remained low.

Conclusions

Implementation of a hybrid HGB service significantly facilitated early LC, nearly doubling the proportion of patients receiving early LC. Although performance improved further as the service matured, the verified overall increase from 13.1% to 30.2% represents the key finding. These results support the feasibility of guideline-aligned, structured HGB pathways within district general hospital settings and provide a foundation for prospective multi-centre validation.

## Introduction

Acute cholecystitis (AC) is an inflammatory disorder of the gallbladder that usually arises from obstruction of the cystic duct, predominantly caused by gallstones. Approximately 10% of patients with symptomatic gallstone disease encounter AC during the course of their condition [1]. In the United Kingdom, gallstone disease affects an estimated 10-15% of the adult population, equating to approximately 5-6 million individuals, although only 1-3% develop symptoms annually. Therefore, AC represents a significant contributor to emergency general surgical admissions within the NHS, with substantial implications for hospital bed occupancy and resource utilisation [2,3].

Laparoscopic cholecystectomy (LC) continues to be the gold standard for treating AC, providing reduced morbidity and mortality rates in comparison to open surgery [4]. Patients are frequently discharged within 24 hours post-surgery, with follow-up appointments arranged after three to four weeks [3]. Nevertheless, certain circumstances may postpone or prevent early LC. These include diagnostic uncertainty, scheduling delays for imaging such as magnetic resonance cholangiopancreatography (MRCP) or endoscopic retrograde cholangiopancreatography (ERCP), admission to medical instead of surgical wards, restricted surgeon availability, and logistical or financial limitations [5]. Daniak et al. (2008) suggested that lLC should ideally be conducted within three days after presentation and advocated for the optimisation of diagnostic paths to prevent unwarranted delays [5]. Evidence-based interventions by the Academy of Medical Royal Colleges also suggest that LC should ideally be conducted within three days of presentation [6].

Emergency LC was markedly more cost-effective, costing less than half the price of elective LC following a single emergency admission and less than one-third of the cost after recurrent admissions. Achieving an 80% early LC target could save the trust approximately £1.9 million annually, while simultaneously improving bed availability and reducing workforce absenteeism [7]. These findings highlight the economic and clinical advantages of prioritising early LC, as demonstrated in previous studies showing that delayed intervention increases readmission rates, prolongs hospital stay, and raises overall healthcare costs. Notably, in the study by Wiggins et al. (2018), 42,620 patients were managed conservatively with 16,088 readmissions (37.7%) [8].

Despite the pandemic era of COVID-19 being three years behind the time of writing, the backlog of waiting list for consultant-led referrals remains high, as reported by the BMJ when compared to the pre-pandemic era, with November 2026 being at 6.17 million patients when compared to 4.39 million at the start of the pandemic [9].

Prolonged wait before surgery is shown to increase morbidity and reduce the overall quality of life in patients [5,10,11]. Somasekar et al. (2002) observed that early laparoscopic surgery reduced the overall costs of treating gallstone disease, as this prevents recurrence of disease warranting emergency admissions [12]. It is noted that the United Kingdom has poor compliance with early LC despite recommendations and guidelines from the National Institute for Health and Care Excellence (NICE) and Association of Upper Gastrointestinal Surgeons (AUGIS), with the service offered by only 11-20% of general surgeons compared to international standards [3,13-15].

The Hot Gallbladder (HGB) service is recommended by the Royal College of Surgeons England (RCSEng) and the Association of Surgeons of Great Britain and Ireland (ASGBI) as a safe, cost-effective method to enhance patient outcomes [14]. The Tokyo 2018 guidelines outlined a flowchart for the management of AC, which suggested that early LC should be performed as soon as possible for patients classed under Grade I (mild) and Grade II (moderate) AC, after assessing risk level of surgery by determining the Charlson Comorbidity Index (CCI) and the American Society of Anesthesiologists (ASA) score for the patient [16].

The World Society of Emergency Surgery (WSES) 2020 also suggested early LC as the standard of care in the treatment of acute calculus cholecystitis, even in subgroups of patients who have a high risk of surgery generally, such as frailty, cardiac and renal disease, and cirrhotic patients [3]. The guidelines outlined in AUGIS 2016 also recommended early LC within the same admission period or in seven days for AC patients, with NICE guidelines 2014 having similar recommendations, for LC to be offered within one week of diagnosis to AC patients [1,14].

In our institution, the HGB service operates as a hybrid pathway, combining two complementary models of care. Eligible patients presenting with acute biliary disease are either managed with early LC during the index admission when theatre capacity permits, or discharged following acute assessment and re-admitted through a fast-tracked outpatient pathway with prioritised imaging, clinic review, and theatre access within five to seven days of presentation. This approach aims to maintain the principles of early surgery while accommodating real-world capacity constraints within a district general hospital setting.

The primary objective of this study was to evaluate the implementation of a hybrid HGB pathway at a district general hospital in the United Kingdom, incorporating both inpatient index admission and fast-tracked outpatient management for eligible patients. It analyses patient outcomes over a 15-month period (January 2023 to March 2024) and service efficiency, focusing on early LC uptake and alignment with national and international guidelines. Secondary objectives were to assess service efficiency indicators, including access to imaging, endoscopy, and theatre capacity; to evaluate postoperative outcomes such as complication and 30-day readmission rates; and to determine the degree of alignment between local practice and national and international guideline recommendations.

## Materials and methods

Study design and setting

This study was a retrospective service evaluation conducted at Walsall Manor Hospital, a 550-bed District General Hospital in the United Kingdom. The HGB pathway was formally introduced in May 2023 to improve access to early LC for patients presenting with acute biliary disease. Data were collected over a 15-month period from January 2023 to March 2024. The pre-implementation phase was January to March 2023, followed by initial implementation (May to July 2023) and expanded implementation (August 2023 to March 2024). As anonymised routine data were used, informed consent and ethical approval were not required. The HGB pathway included both inpatient and expedited outpatient components. Patients admitted with suspected or confirmed AC were initially reviewed in the Surgical Acute Care Unit (SACU). Eligible patients were either listed for surgery during the same admission (inpatient HGB) or discharged with a scheduled outpatient ultrasound and follow-up appointment (outpatient HGB) to ensure LC within five days of admission or within seven days of symptom onset. This model aimed to maintain continuity of acute surgical care while optimising resource use in a district general hospital setting.

Description of the Hot Gallbladder pathway

The HGB service operates as a hybrid model, incorporating both inpatient and expedited outpatient pathways. Patients presenting to the SACU with suspected AC were reviewed by the on-call general surgical team. Eligibility for the HGB pathway was assessed at the point of admission based on predefined clinical and radiological criteria.

Inpatient Pathway

Patients deemed suitable and for whom theatre capacity was available were listed for LC during the same admission.

Outpatient Pathway

When same-admission surgery was not feasible due to capacity constraints, clinically stable patients were discharged and enrolled into the HGB outpatient pathway. These patients were scheduled for dedicated ultrasound scanning within 72 hours, rapid-access HGB clinic review, and prioritised placement on dedicated HGB theatre lists, with the aim of performing surgery within five days of admission or within seven days of symptom onset.

Theatre, Clinic, and Imaging Resources

The service was supported by two dedicated all-day HGB theatre lists per week, six rapid-access HGB outpatient clinic slots per week, and three reserved ultrasound slots allocated specifically to HGB patients.

Inclusion and exclusion criteria

All adult patients (≥18 years) presenting to the SACU with right upper quadrant pain or suspected biliary disease were screened for eligibility. Patients were considered suitable for the HGB pathway if they met predefined clinical and radiological criteria. These included body mass index (BMI) <40 kg/m², ASA grade I-II, symptom duration ≤7 days, absence of common bile duct (CBD) dilatation on initial ultrasound or CT imaging, and no history of major upper abdominal surgery.

Exclusion criteria included severe deranged liver function tests (LFTs) with CBD stones, acalculous cholecystitis, gallbladder polyps, ASA grade ≥IV, pregnancy, or age <18 years. While the Tokyo 2018 guidelines do not specify ASA classifications, patients with a BMI ≥40 kg/m² are generally considered at increased perioperative risk and may be unsuitable for early LC [14]. However, selected patients with BMI ≥40 kg/m² and ASA grade III were considered on an individual basis, provided they demonstrated adequate functional capacity, defined as a Duke Activity Status Index score >34, following consultant anaesthetic assessment.

Baseline LFTs were reviewed at presentation. Patients with normal LFTs proceeded directly through the HGB pathway. In cases of isolated bilirubin or alkaline phosphatase elevation, further evaluation with MRCP was undertaken to exclude CBD stones. Patients were included in the operative pathway only after imaging confirmed the absence of choledocholithiasis.

When an AC patient presented through the SACU and was eligible for the HGB list, an elective HGB ultrasound was requested, with the patient returning for an outpatient appointment. The ultrasound results were explained to the patient during the appointment, and if patients met the eligibility criteria (no evidence of CBD stones), they then consented for LC. Once consent was obtained, patients were prepared for LC. For example, patients with a BMI >30 kg/m² should abide by a liver-shrinkage diet. Before LC, patients were provided with information via leaflets and the medications were reviewed.

Data collection and verification

A consecutive sampling method included all eligible patients within the study period. A total of 286 patients were identified, with 78 presenting with AC and the remainder with biliary colic or gallstone pancreatitis. Data sources included SACU admission records, the HGB Microsoft Teams list, radiology databases, theatre logs, and electronic patient records. Collected variables included demographics, diagnosis, imaging results, timing of LC, MRCP/ERCP intervals, postoperative outcomes, and readmissions. “Early LC” was defined as LC performed within five days of admission or within seven days of symptom onset, in accordance with NICE and AUGIS standards.

Outcomes

The primary outcome was the proportion of patients undergoing early LC, defined as surgery performed during the index admission or within seven days of symptom onset. Secondary outcomes included time to surgery, access to imaging and ERCP, postoperative complications, and 30-day readmission rates.

Data analysis

Data were analysed using IBM SPSS Statistics version 29.0 (IBM Corp., Armonk, NY, USA). Descriptive statistics summarised demographic and procedural data. Continuous variables were reported as mean ± standard deviation or median (interquartile range), and categorical variables as n (%). Differences between pre- and post-HGB cohorts were evaluated using independent t-tests or Mann-Whitney U tests for continuous data, and chi-square or Fisher’s exact tests for categorical data. A p-value <0.05 was considered statistically significant. Visual figures were prepared using GraphPad Prism 10. Data validation followed STROBE guidelines with dual-entry verification [17]. All datasets were cross-checked to confirm accuracy of cohort allocation, diagnosis, operative timing, and outcomes before analysis.

## Results

A total of 286 patients were analysed, with a mean age of 45.0 ± 12.4 years and a female predominance (n = 230, 80.4%). Table 1 presents the demographic and diagnostic distribution. The most common reason for patients attending SACU for right upper quadrant pain was biliary colic, followed by AC and gallstone pancreatitis. Following implementation of the HGB pathway, the overall proportion of patients undergoing early LC increased to 30.2%, compared with 13.1% pre-HGB (p < 0.01), as shown in Table 2. Notably, the service achieved a peak rate of 56.6% during the final quarter of the study period, demonstrating the pathway’s potential once fully operational. There was a 16.3% reduction in patients not undergoing early LC due to scheduling into elective lists.

**Table 1:** Baseline characteristics. No statistically significant baseline differences between pre- and post-HGB patients. χ² = Pearson chi-square; t = independent samples t-test; U = Mann–Whitney test statistic. Fisher’s exact test was used where the expected cell count was <5. Statistical significance was defined as p < 0.05. HGB = Hot Gallbladder

Variable	Overall (n = 286)	Pre-HGB (n = 84)	Post-HGB (n = 202)	Test statistic; p-value
Age, years (mean ± SD)	45.0 ± 12.4	44.2 ± 11.9	45.3 ± 12.7	t(284) = 0.64; p = 0.52
Female, n (%)	230 (80.4)	66 (78.6)	164 (81.2)	χ²(1) = 0.25; p = 0.62
Acute cholecystitis, n (%)	78 (27.3)	22 (26.2)	56 (27.7)	χ²(1) = 0.05; p = 0.83
Biliary colic, n (%)	178 (62.2)	54 (64.3)	124 (61.4)	χ²(1) = 0.22; p = 0.64
Gallstone pancreatitis, n (%)	30 (10.5)	8 (9.5)	22 (10.9)	χ²(1) = 0.09; p = 0.77

**Table 2:** Early LC by period with the chi-square test. Significant increase in early LC rate after HGB pathway implementation. LC = laparoscopic cholecystectomy; HGB = Hot Gallbladder

Outcome	Overall	Pre-HGB (n = 84)	Post-HGB (n = 202)	Test statistic; p-value
Early LC, n (%)	72 (25.2)	11 (13.1)	61 (30.2)	χ²(1) = 9.19; p = 0.0024

The mean MRCP-to-ERCP interval decreased significantly from 28.1 days (median = 20.5) to 14.1 days (median = 0.5) (p = 0.03) (Table 3). The weekly demand averaged 1.95 MRCPs and 0.56 ERCPs. Two LCs were cancelled due to deranged LFTs. Only one (0.3%) patient was readmitted for postoperative pain, with no conversions to open surgery or mortality.

**Table 3:** Imaging and endoscopy turnaround times. Values are presented as mean (median). The Mann–Whitney U test was used for non-parametric distributions. Median turnaround times for imaging and surgery were significantly reduced post-HGB (p < 0.05). LC = laparoscopic cholecystectomy; ERCP = endoscopic retrograde cholangiopancreatography; MRCP = magnetic resonance cholangiopancreatography; HGB = hybrid Hot Gallbladder

Interval (days)	Pre-HGB	Post-HGB	Test statistic; p-value
MRCP → ERCP	28.1 (20.5)	14.1 (0.5)	U = 41.5; p = 0.03
Admission → LC	5.6 (4.0)	3.2 (2.0)	U = 48.0; p = 0.04

During the implementation of the HGB list, it was noted that per week, approximately 1.95 MRCP procedures would need to happen alongside approximately 0.56 ERCP procedures to be performed per week (Table 4). Two early LCs were cancelled due to deranged LFTs. During the 15 months of the HGB list, one patient developed complications and was re-admitted due to non-specific postoperative abdominal pain in November.

**Table 4:** Weekly service demand for imaging and endoscopy among HGB patients. Approximately 2 MRCP and 0.5 ERCP procedures per week were required to sustain HGB throughput. ERCP = endoscopic retrograde cholangiopancreatography; MRCP = magnetic resonance cholangiopancreatography; HGB = hybrid Hot Gallbladder

Modality	Mean per week	Median per week
MRCP	1.95	2
ERCP	0.56	1

## Discussion

The introduction of the HGB service at our institution significantly improved compliance with national and international recommendations for early LC. The increase in early LC rates and the marked reduction in diagnostic-to-intervention intervals demonstrate the effectiveness of structured service redesign. These results align closely with other published data showing that early LC within the index admission is both clinically advantageous and economically sustainable [5,12-14].

Timely surgical intervention in AC reduces complications such as gallbladder perforation, empyema, and recurrent biliary events, while avoiding the morbidity associated with repeated hospital admissions. The primary outcome of this study was a statistically significant increase in early LC rates from 13.1% to 30.2% following implementation of the HGB pathway (p = 0.0024). This demonstrates the impact of structured, guideline-based service delivery on improving timely surgical intervention for acute biliary disease. The higher rate achieved in the final quarter (56.6%) illustrates the progressive optimisation of the service rather than the overall mean performance. This finding is consistent with national data reporting improvements from 20% to more than 60% following the establishment of dedicated HGB pathways [12,14].

Beyond clinical outcomes, the economic implications of early LC are substantial. Emergency LC incurs a mean cost of approximately £2,053, compared to £5,661 for elective LC following a single emergency admission, and £7,453 for patients undergoing multiple recurrent admissions. Consequently, achieving an 80% early LC target could yield an estimated annual saving of nearly £1.9 million for a medium-sized district general hospital, according to the literature [7]. These savings could be reinvested into maintaining a dedicated HGB service, reducing inpatient bed occupancy, and improving staff resource allocation through fewer repeat admissions. The wider benefits include decreased patient morbidity, shorter waiting lists, and reduced workforce absenteeism due to delayed treatment and prolonged recovery [18].

Our findings further highlight that a multidisciplinary, protocol-driven approach, incorporating early ultrasound access, daily HGB clinics, and reserved theatre lists, streamlines the management of acute gallbladder disease. Gurusamy et al. [19] also confirmed through a meta-analysis that early LC is associated with lower overall healthcare expenditure and comparable or lower postoperative morbidity relative to delayed surgery.

While the clinical and economic benefits are clear, barriers to consistent early LC remain. These include limited emergency theatre capacity, variability in surgical expertise, and delays in diagnostic imaging, particularly MRCP. The current HGB model addresses these constraints through dedicated imaging slots and prioritised surgical scheduling. Implementation at scale across other district general hospitals could further reduce the national backlog of gallbladder surgery, which remains a significant challenge for the NHS post-pandemic [2,9].

Limitations and strengths

This study has several important limitations. First, its retrospective design limits causal inference and introduces the potential for selection and information bias. Second, the study was conducted at a single district general hospital, which may restrict generalisability to institutions with differing patient demographics, theatre capacity, or access to imaging and endoscopy services. Third, the HGB service operated as a hybrid pathway incorporating both index-admission and expedited outpatient surgery; while this reflects real-world practice within the NHS, it introduces heterogeneity that may affect reproducibility across centres with alternative service structures. Fourth, outcome data were derived from routinely collected clinical records, which may be subject to documentation variability and incomplete capture of postoperative events occurring outside the institution.

Despite these limitations, the study has notable strengths. It evaluated a clinically relevant service intervention addressing a well-recognised national challenge in delivering timely LC. The pre- and post-implementation design was appropriate for assessing the impact of service redesign and reflects pragmatic conditions under which NHS pathway changes are introduced. Data were cross-checked across multiple hospital systems to improve accuracy, and outcome measures, including early LC rates and 30-day readmission, were objective, clinically meaningful, and aligned with national guideline standards. Importantly, this study provides real-world evidence supporting the feasibility of implementing a structured HGB pathway within a resource-limited district general hospital and establishes a foundation for future prospective, multi-centre validation.

## Conclusions

Implementation of a structured HGB pathway was associated with a statistically significant increase in early LC from 13.1% to 30.2%. While this improvement demonstrates enhanced access to timely surgery, the findings should be interpreted in the context of the study’s retrospective design, single-centre setting, and hybrid pathway structure. The results support the feasibility of service reconfiguration to improve guideline adherence but do not establish causality or system-wide outcome benefit. Further prospective, multi-centre evaluation is required to validate these findings and assess long-term clinical and economic impact.
